# The UDP-Glycosyltransferase Family in *Drosophila melanogaster*: Nomenclature Update, Gene Expression and Phylogenetic Analysis

**DOI:** 10.3389/fphys.2021.648481

**Published:** 2021-03-17

**Authors:** Seung-Joon Ahn, Steven J. Marygold

**Affiliations:** ^1^Department of Biochemistry, Molecular Biology, Entomology and Plant Pathology, Mississippi State University, Starkville, MS, United States; ^2^FlyBase, Department of Physiology, Development and Neuroscience, University of Cambridge, Cambridge, United Kingdom

**Keywords:** *Drosophila melanogaster*, UDP-glycosyltransferase, UGT, nomenclature, detoxification, conjugation

## Abstract

UDP-glycosyltransferases (UGTs) are important conjugation enzymes found in all kingdoms of life, catalyzing a sugar conjugation with small lipophilic compounds and playing a crucial role in detoxification and homeostasis. The UGT gene family is defined by a signature motif in the C-terminal domain where the uridine diphosphate (UDP)-sugar donor binds. UGTs have been identified in a number of insect genomes over the last decade and much progress has been achieved in characterizing their expression patterns and molecular functions. Here, we present an update of the complete repertoire of UGT genes in *Drosophila melanogaster* and provide a brief overview of the latest research in this model insect. A total of 35 UGT genes are found in the *D. melanogaster* genome, localized to chromosomes 2 and 3 with a high degree of gene duplications on the chromosome arm 3R. All *D. melanogaster* UGT genes have now been named in FlyBase according to the unified UGT nomenclature guidelines. A phylogenetic analysis of UGT genes shows lineage-specific gene duplications. Analysis of anatomical and induced gene expression patterns demonstrate that some UGT genes are differentially expressed in various tissues or after environmental treatments. Extended searches of UGT orthologs from 18 additional *Drosophila* species reveal a diversity of UGT gene numbers and composition. The roles of *Drosophila* UGTs identified to date are briefly reviewed, and include xenobiotic metabolism, nicotine resistance, olfaction, cold tolerance, sclerotization, pigmentation, and immunity. Together, the updated genomic information and research overview provided herein will aid further research in this developing field.

## Introduction

UDP-glycosyltransferases (UGTs) are a superfamily of enzymes found in all kingdoms of life, including animals, plants, fungi, bacteria, and some viruses (Bock, 2016). UGTs catalyze the covalent addition of sugars from uridine diphosphate (UDP) sugar donors to a broad range of lipophilic small molecules, playing a crucial role in conjugation, detoxification and elimination of exogenous and endogenous toxic compounds, as well as in regulation and distribution of endogenous signal molecules and metabolites (Meech et al., 2019). Mammalian UGTs were previously called “UDP-glucuronosyltransferases” as most research articles in drug metabolism dealt with enzymes that mainly use UDP-glucuronic acid as the sugar donor; however, the UGT Nomenclature Committee recommended the use of “UDP-glycosyltransferase” in order to include enzymes that do not use UDP-glucuronic acid (Mackenzie et al., 2005). The same notion has been adopted for non-mammalian UGTs (Meech et al., 2012), including insects as they predominantly use UDP-glucose as the sugar donor (Myers and Smith, 1954; Dutton and Ko, 1964; Ahmad and Forgash, 1976; Kramer and Hopkins, 1987; Rausell et al., 1997; Wang et al., 1999).

The first evidence of UGT activity in insects was obtained by a chromatographic analysis of m-aminophenyl glucoside from feces of a locust, *Locusta migratoria*, suggesting insects conjugate the hydroxyl compounds with glucose, instead of glucuronic acid (Myers and Smith, 1954). Biochemical studies in a variety of insect species indicated that the glucose conjugation plays an important role in diverse physiological processes in insects, such as detoxification (Smith, 1955; Wilkinson, 1986; Ahn et al., 2011), sclerotization (Kramer and Hopkins, 1987; Hopkins, 1992), pigmentation (Hopkins and Ahmad, 1991; Wiesen et al., 1994), and insecticide resistance (Lee et al., 2005). Molecular studies revealed that a UGT is responsible for the glycosylation of flavonoids in the silkworm cocoon (Daimon et al., 2010). Antenna-specific UGTs were detected by gene expression analysis in a moth, *Spodoptera littoralis*, suggesting specific roles in olfaction (Bozzolan et al., 2014). It was revealed that benzoxazinoids, the indole-derived plant defense compounds, are stereoselectively inactivated by UGT enzymes in the fall armyworm, *Spodoptera frugiperda* (Israni et al., 2020). Also, some UGTs were shown to be associated with insecticide resistance (Li et al., 2017; Chen et al., 2019, 2020; Zhou et al., 2019; Pan et al., 2020). Several UGTs have been identified and characterized in the *Drosophila* genus, with a focus on the model organism *D. melanogaster*. *Drosophila* UGTs have been shown to function in diverse processes including xenobiotic metabolism, nicotine resistance, olfaction, cold tolerance, sclerotization, pigmentation, and immunity (summarized in Table 1). Among non-insect arthropods, the two-spotted spider mite, *Tetranychus urticae*, has been intensively studied for the substrate specificity of its UGTs (Snoeck et al., 2019), which are most likely acquired from bacteria via horizontal gene transfer (Ahn et al., 2014).

**TABLE 1:** Summary of UGT functions in *Drosophila melanogaster* and related species.

Species	UGT gene	Function	References
**Xenobiotic metabolism**			
*D. melanogaster*	*unknown*	Some standard xenobiotic substrates (4-nitrophenol, 1-naphthol, and 2-naphthol) were glucosylated by adult crude homogenates, the first enzymatic study.	Real et al., 1991
*D. melanogaster*	*unknown*	FPLC-aided enzyme fractions showed UGT activities toward the two xenobiotic substrates (1-naphthol and 2-naphthol) in different developmental stages, suggesting the existence of multiple UGT isoenzymes.	Rausell et al., 1997
*D. melanogaster*	*Ugt37A1*	UGT37A1 protein was expressed in *Sf*21 cells and tested toward 38 compounds, but no activity was detected.	Luque and O’Reilly, 2002
**Nicotine resistance**			
*D. melanogaster*	*Ugt35C1*	QTL mapping, RNA-Seq, RNAi and CRISPR/Cas9-mediated knock-out experiments confirmed that *Ugt35C1* (named *Ugt86Dd* in the paper) is associated with nicotine resistance.	Marriage et al., 2014; Highfill et al., 2017; Macdonald and Highfill, 2020
**Olfaction**			
*D. melanogaster*	*Ugt35B1*	Among the 5 UGT genes first ever sequenced in insect, *Ugt35B1* showed a high gene expression level in antennae.	Wang et al., 1999
*D. melanogaster*	*Ugt35B1, Ugt35A1, Ugt37D1, Ugt302C1*	Along with *Ugt35B1*, three additional UGT genes (*Ugt35A1, Ugt37D1*, and *Ugt302C1*) were highly expressed in antennal transcriptome.	Younus et al., 2014
*D. melanogaster*	*Ugt36E1*	*Ugt36E1* expressed in antennal olfactory sensory neurons is involved in pheromone detection, revealed by UAS-Gal4 mutation and RNAi methods.	Fraichard et al., 2020
**Cold tolerance**			
*D. ananassae*	*Ugt301D1*	Cold shock led to a downregulation of *Ugt301D1* (GF15058 in *D. ananassae*) in the cold-sensitive strains, but not in the cold-tolerant strains. *D. melanogaster Ugt301D1* was also downregulated after cold shock.	Königer and Grath, 2018
**Sclerotization**			
*D. melanogaster*	*unknown*	*N*-acetyldopamine, as a sclerotizing agent of the insect cuticle, was found in a form of glucoside in many insects, including *D. melanogaster.*	Okubo, 1958
*D. busckii*	*unknown*	Tyrosine was rapidly accumulated as a glucoside conjugate in the last instar larvae and then suddenly disappeared at pupae of *D. busckii*, suggesting that the tyrosine glucoside serves as a tyrosine reservoir for the sclerotization of the pupal exoskeleton. (Other species including *D. melanogaster* predominantly forms tyrosine phosphate instead of glucoside)	Chen et al., 1978
**Pigmentation**			
*D. melanogaster*	*unknown*	Xanthurenic acid glucoside was accumulated in some eye-color mutants of *D. melanogaster*.	Ferré et al., 1985
*Drosophila* spp.	*unknown*	Xanthurenic acid glucoside was detected mostly in the *Sophophora* subgenus from a wide range survey of 29 *Drosophila* species.	Real and Ferré, 1989
*Drosophila* spp.	*unknown*	Enzymatic activity responsible for the conjugation of xanthurenic acid was measured with crude homogenates of various *Drosophila* species.	Real and Ferré, 1990
**Immunity**			
*D. melanogaster*	*Ugt36A1*	*Ugt36A1* (originally named *Dorothy*) was detected in the lymph glands and pericardial cells. *Dorothy*-Gal4 transgenic flies were constructed for studying the role of cellular immune system and melanization.	Rodriguez et al., 1996; Zhou et al., 2001; Kimbrell et al., 2002

During the last two decades, genome and transcriptome sequencing of insects has generated genome-wide analyses of UGT genes in a variety of insects (Luque and O’Reilly, 2002; Huang et al., 2008; Ahn et al., 2012; Hu B. et al., 2019), revealing that the UGT gene family comprises multiple genes in each species, ranging from 12 (honeybee) to 58 (aphid) (Ahn et al., 2012). Given these and similar studies of non-insect genomes, the UGT Nomenclature Committee was formed to assign systematic names to the large number of UGTs, defining the families (e.g., UGT36) and subfamilies (e.g., UGT36A) at >45% and >60% amino acid sequence identity, respectively^1^. Originally, families 1–50 are reserved for animals, 51–70 for fungi and yeasts, 71–100 for plants, and 101–200 for bacteria; if these number assignments become depleted, the family number increases by 10-fold (Mackenzie et al., 1997). For insects and insect viruses, the UGT family numbers have been assigned from 31 to 50, resuming in the range 301–500 (Ahn et al., 2012).

As a model insect, it is particularly important that the UGT genes of *D. melanogaster* are identified and named in accordance with the UGT Nomenclature Committee guidelines; these genes define the range of insect UGT family numbers, and also provide a consensus standard to study UGT genes from other insects that will be annotated in the future. For this purpose, we report here the complete repertoire of *D. melanogaster* UGT genes with updated nomenclature, genomic architecture and gene expression data. We also identify orthologous genes from 18 additional *Drosophila* species in order to view the *D. melanogaster* UGTs from an evolutionary perspective.

## Results

### *D. melanogaster* UGT Nomenclature

The first *Drosophila melanogaster* UGT gene to be identified, *Dorothy* (currently *Ugt36A1*), was named after a character of *The Wizard of Oz* (Rodriguez et al., 1996). A little later, five other *D. melanogaster* UGT genes, *Ugt35a*, *Ugt35b*, *Ugt37a1*, *Ugt37b1*, and *Ugt37c1* (lowercase letters were initially used to indicate subfamily membership), were among the first UGT genes to be named in consultation with the UGT Nomenclature Committee (Wang et al., 1999). Subsequently, several other *D. melanogaster* UGTs were directly named in FlyBase according to their cytogenetic locations (e.g., *Ugt36Ba – Ugt36Bc*, *Ugt58Fa*, and *Ugt86Da* – *Ugt86Dj*) (Table 2), which is evidently confusing given the superficial resemblance between this notation and the UGT Committee nomenclature. Ahn et al. (2012) revised and curated the *D. melanogaster* UGTs, employing the systematic names to maintain consistency with the universal nomenclature and the five previously assigned official names. In the current study, we have completed the list of *D. melanogaster* UGT genes and have updated the gene symbols and names within FlyBase to adopt the systematic nomenclature. Furthermore, we have added a UGT “gene group” page to FlyBase that conveniently lists all these genes in a single report to facilitate further analysis and download of associated data^2^.

**TABLE 2:** *D. melanogaster* UGT gene nomenclature and genomic data.

Family	Sub-family	FlyBase symbol	Synonym	CG no.	Genomic coordinates	Cyto. location	No. introns	Protein length (aa)
UGT35	35A	*Ugt35A1*	*Ugt35a*	CG6644	3R:11170817..11172664 (−)	86D5	1	537
	35B	*Ugt35B1*	*Ugt35b*	CG6649	3R:11168503..11170246 (−)	86D5	1	516
	35C	*Ugt35C1*	*Ugt86Dd*	CG6633	3R:11126597..11128328 (−)	86D4	1	517
	35D	*Ugt35D1*	*–*	CG31002	3R:31393582..31395304 (−)	100C3	1	521
	35E	*Ugt35E1*	*Ugt86Dg*	CG17200	3R:11164423..11166074 (−)	86D5	1	527
		*Ugt35E2*	*Ugt86De*	CG6653	3R:11166177..11167981 (−)	86D5	1	527
UGT36	36A	*Ugt36A1*	*Dot*	CG2788	2L:3619097..3621573 (+)	24A1-2	2	537
	36D	*Ugt36D1*	*–*	CG17323	2L:18823548..18826716 (+)	37B1	3	519
	36E	*Ugt36E1*	*–*	CG17322	2L:18826770..18829059 (+)	37B1	2	517
	36F	*Ugt36F1*	*–*	CG17324	2L:18819344..18822573 (+)	37B1	4	525
UGT37	37A	*Ugt37A1*	*–*	CG11012	2L:20372409..20374104 (−)	38C5	1	525
		*Ugt37A2*	*–*	CG5724	3R:12739642..12741417 (+)	87C8	1	530
		*Ugt37A3*	*–*	CG5999	3R:12741958..12743680 (−)	87C8	1	530
	37B	*Ugt37B1*	*–*	CG9481	2L:6225048..6226842 (+)	26B11	1	537
	37C	*Ugt37C1*	*–*	CG8652	2R:16843296..16845038 (−)	53D12	0	525^1)^
		*Ugt37C2*	*Ugt36Ba*	CG13270	2L:16794211..16796009 (+)	36B1	0	523
	37D	*Ugt37D1*	*Ugt36Bc*	CG17932	2L:16799025..16801584 (+)	36B1	1	543
	37E	*Ugt37E1*	*Ugt36Bb*	CG13271	2L:16796595..16798273 (+)	36B1	1	539
UGT49	49B	*Ugt49B1*	*–*	CG4302	2R:21212880..21214972 (−)	57D1-2	3	532
		*Ugt49B2*	*–*	CG6475	3R:21397781..21399742 (−)	93D10-E1	3	526
	49C	*Ugt49C1*	*–*	CG15661	2R:21215435..21217779 (−)	57D2	4	530
UGT50	50B	*Ugt50B3*	*–*	CG30438	2R:5496674..5549543 (+)	41F2-3	5	435, 524^2)^
UGT301	301D	*Ugt301D1*	*–*	CG10178	2L:18509560..18513512 (+)	36F6	2	530
UGT302	302C	*Ugt302C1*	*Ugt86Da*	CG18578	3R:11157098..11159744 (+)	86D5	2	528
	302E	*Ugt302E1*	*Ugt86Dc*	CG4739	3R:11154626..11156513 (+)	86D5	1	521
	302K	*Ugt302K1*	*Ugt86Di*	CG6658	3R:11151026..11153849 (−)	86D5	2	519
UGT303	303A	*Ugt303A1*	*Ugt86Dh*	CG4772	3R:11175529..11177910 (+)	86D6	1	526
	303B	*Ugt303B1*	*–*	CG16732	3R:23534900..23536710 (−)	95A1	1	516, 519^2)^
		*Ugt303B2*	*–*	CG10168	3R:23536993..23538908 (−)	95A1	1	540
		*Ugt303B3*	*–*	CG10170	3R:23532945..23534767 (−)	95A1	1	539
UGT304	304A	*Ugt304A1*	*Ugt86Dj*	CG15902	3R:11173441..11175408 (−)	86D5-6	1	529
UGT305	305A	*Ugt305A1*	*–*	CG18869	3L:4059770..4061923 (+)	64A5	3	583
UGT307	307A	*Ugt307A1*	*–*	CG11289	2L:7067983..7069546 (+)	27D7-E1	1	502
UGT316	316A	*Ugt316A1*	*–*	CG3797	3L:19059400..19062816 (−)	75F6	3	636
UGT317	317A	*Ugt317A1*	*Ugt58Fa*	CG4414	2R:22641786..22643917 (−)	58F3	3	529

*^1)^485 aa in FlyBase (FB2020_05) but manually amended here, ^2)^alternative splicing.*

### Genomic Distribution of UGT Genes

**FIGURE 1:**
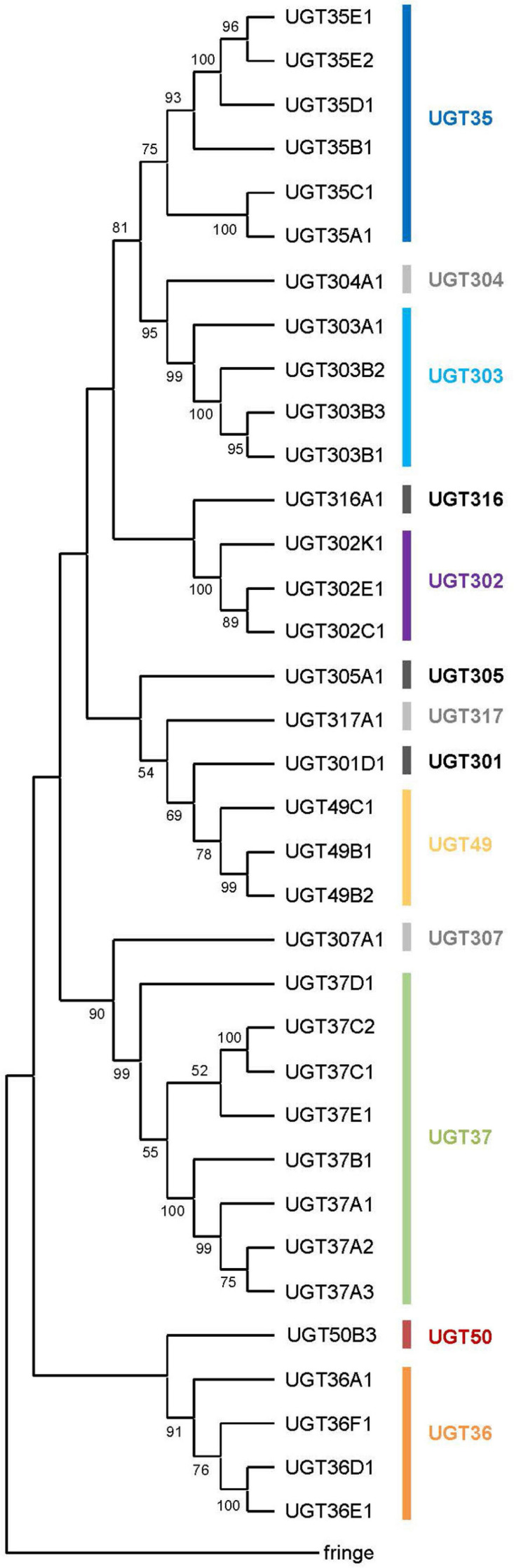
A phylogenetic tree of the UDP-glycosyltransferases from *Drosophila melanogaster*. All the 35 UGT protein sequences and the fringe protein sequence (as an outgroup) were aligned using ClustalW and a consensus phylogenetic tree was constructed using the Maximum Likelihood method and JTT matrix-based model. The percentage of replicate trees in which the associated taxa clustered together in the bootstrap test (1,000 replicates) are shown next to the branches (Those less than 50% are omitted). Evolutionary analyses were conducted in MEGA X.

Wang et al. (1999) identified 9–10 putative UGT gene sequences, including the five named ones (see above), from cDNA libraries and the incomplete genome databases available at the time. Upon completion of the *D. melanogaster* genome (Adams et al., 2000), the first genome-wide annotation of multiple UGT genes was conducted and a total of 33 putative UGT genes were reported together with a phylogenetic and genomic analysis (Luque and O’Reilly, 2002). Ahn et al. (2012) revised the sequences in detail and identified an additional gene (*Ugt50B3*). The current study has added one further gene (*Ugt305A1*), resulting in a complete repertoire of 35 UGT genes in *D. melanogaster* (Table 2). They are grouped into 13 families according to the nomenclature system: UGT35 (6 genes), UGT36 (4 genes), UGT37 (8 genes), UGT49 (3 genes), UGT50 (1 gene), UGT301 (1 gene), UGT302 (3 genes), UGT303 (4 genes), and 1 gene in each of UGT304, UGT305, UGT307, UGT316, and UGT317 (Table 2 and Figure 1).

All 35 UGT genes are found on the two major autosomes (chromosome 2 with 16 genes and chromosome 3 with 19 genes); none are located on the minor autosome (chromosome 4) or the sex chromosomes (Table 2 and Supplementary Figure 1). Among different chromosomal arms, about half (17 UGT genes) lie on 3R (the right arm of chromosome 3), followed by 2L (11 UGT genes), 2R (5 genes) and 3L (2 genes). A large cluster of UGT genes is found on 3R at the cytogenetic location of 86D4 – 86D6, where ten closely related UGT genes are positioned in tandem. The other multiplied gene families are found in one or two genomic locations in close proximity, whereas the members of another large family, UGT37, are spread across three different chromosomal arms (five in 2L, one in 2R, and two in 3R) (Table 2 and Supplementary Figure 1). It is noteworthy that 3L harbors only two UGT genes (*Ugt305A1* and *Ugt316A1*), both of which seem to be unique in their sequences, and are unusually long (Table 2).

### UGT Gene Structure

All 35 UGT genes are interrupted by intron(s) except for *Ugt37C1* and *Ugt37C2* (Table 2). These two intron-less genes do not seem to originate from bacterial UGT genes due to their sequence similarity to animal UGTs (see Ahn et al., 2014). *D. melanogaster* UGT genes are composed of one to six exons: a majority of genes (19 genes; 54%) comprise 2 exons and the rest of genes have 1, 3, 4 or 5 exons, except one gene (*Ugt50B3*) has 6 exons in its coding sequence (Table 2 and Supplementary Figure 2). The lengths of intron sequences are mostly within the range of 48–85 bp (41 introns) or 108–584 bp (14 introns). Exceptionally, *Ugt50B3* is interrupted by three long introns (1,389, 1,0432, and 8,198 bp) followed by two short ones (63 and 52 bp) (Supplementary Table 1 and Supplementary Figure 3). This, together with the fact it is phylogenetically distinguished from the others (Figure 1) and highly conserved in insects in general (Ahn et al., 2012), suggests *Ugt50B3* is one of the oldest UGT genes.

Splicing variants are found in two UGT genes, *Ugt50B3* and *Ugt303B1*, where two alternative transcripts have been reported (Table 2). The *Ugt50B3* variant is annotated to have an alternative start codon in the middle of what is otherwise the third exon, producing a protein that is 89 amino acids (aa) shorter than the normal one. The *Ugt303B1* variants seem to be derived from alternative splicing sites at the 3’-end of the first exon, resulting in a difference of only 9 nucleotides (3 aa) (Table 2).

The average length of *D. melanogaster* UGT proteins is 532 aa with two outliers, Ugt305A1 (583 aa) and Ugt316A1 (636 aa), which, as noted above, are phylogenetically unique and located in different genomic positions from the other UGT genes. All the UGTs contain an N-terminal signal peptide and a C-terminal transmembrane (TM) domain (Table 2 and Supplementary Figure 4), indicating that the *D. melanogaster* UGTs are located in the endoplasmic reticulum (ER) with their catalytic domains facing the ER lumen, as shown in other animals (Meech et al., 2012). The UGT-defining 44-aa signature sequence in the C-terminal domain, which is predicted to be intimately involved in the binding of UDP-sugar (Meech et al., 2019), is well conserved across the 35 UGTs (Supplementary Figure 5). However, variations shown in some residues in the signature sequence imply different specificity to different sugar donors other than UDP-glucose.

### Phylogenetic Analysis

A consensus Maximum-likelihood tree constructed with deduced amino acid sequences revealed lineage-specific gene amplifications in several families such as UGT35, UGT36, UGT37, UGT49, UGT302, and UGT303 (Figure 1). For example, upon divergence from a common ancestor with *Ugt307A1*, UGT37 seems to have diversified into the largest gene family in *D. melanogaster* UGTs. It is noteworthy that the UGT37 members are spread across five different genomic locations. On the other hand, other multiplied UGTs are most likely diversified by tandem gene duplications, as they are found in the same genomic scaffolds in close proximity (Supplementary Figure 1).

### UGT Gene Expression

**FIGURE 2:**
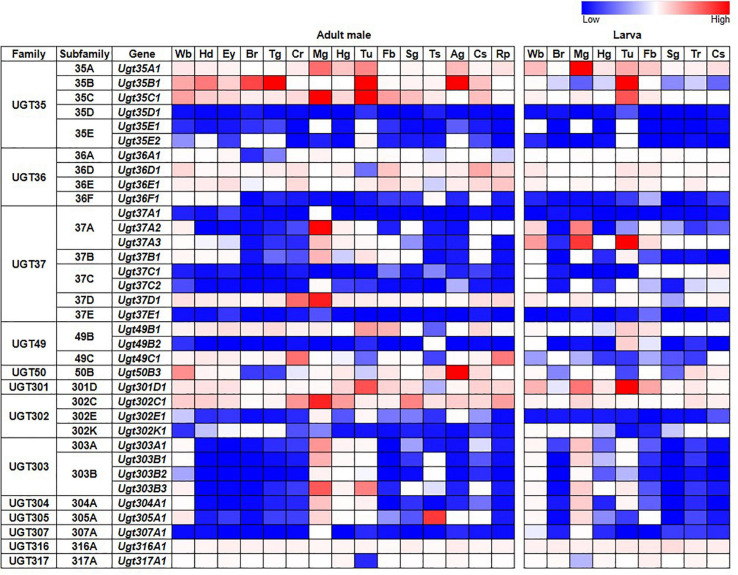
Expression of *D. melanogaster* UGT genes in different tissues of adult males and larvae (FlyAtlas2, Leader et al., 2018). Wb: whole body; Hd: head; Ey: eye; Br: brain/CNS; Tg: thoracicoabdominal ganglion; Cr: crop; Mg: midgut; Hg: hindgut; Tu: Malpighian tubules; Fb: fat body; Sg: salivary gland; Ts: testis; Ag: accessory glands; Cs: carcass; Rp: rectal pad; Tr: trachea. See Supplementary Table 2 for details and equivalent data for adult females.

Tissue-specific expression patterns of *D. melanogaster* UGT genes were analyzed previously by Ahn et al. (2012) using microarray data present in FlyAtlas (Chintapalli et al., 2007). Here, we have revisited this analysis using the higher quality RNAseq data available from the FlyAtlas2 database (Leader et al., 2018) – full data for adult males, adult females and larvae are included in Supplementary Table 2; representative data for adult males and larvae are in Figure 2. UGTs from each family are expressed in every adult and larval tissue at some level. Some UGT genes belonging to multi-gene families (*Ugt35D1* and *Ugt37E1*) are undetectable in any tissue, while several others are expressed only in restricted patterns. In contrast, many UGT genes appear to be expressed ubiquitously, with high expression levels often seen within the digestive and excretory systems, particularly for members of the UGT35 and UGT37 families. Across all UGTs, the highest expression is seen within the adult midgut and larval Malpighian tubules. Of note, *Ugt50B3*, the sole representative of the UGT50 family, shows unusually high expression within the male accessory gland and the female spermatheca, whereas *Ugt305A1* is only expressed at appreciable levels in the testis. Such restricted expression patterns suggest particularly important roles of *Ugt50B3* and *Ugt305A1* within these tissues.

**FIGURE 3:**
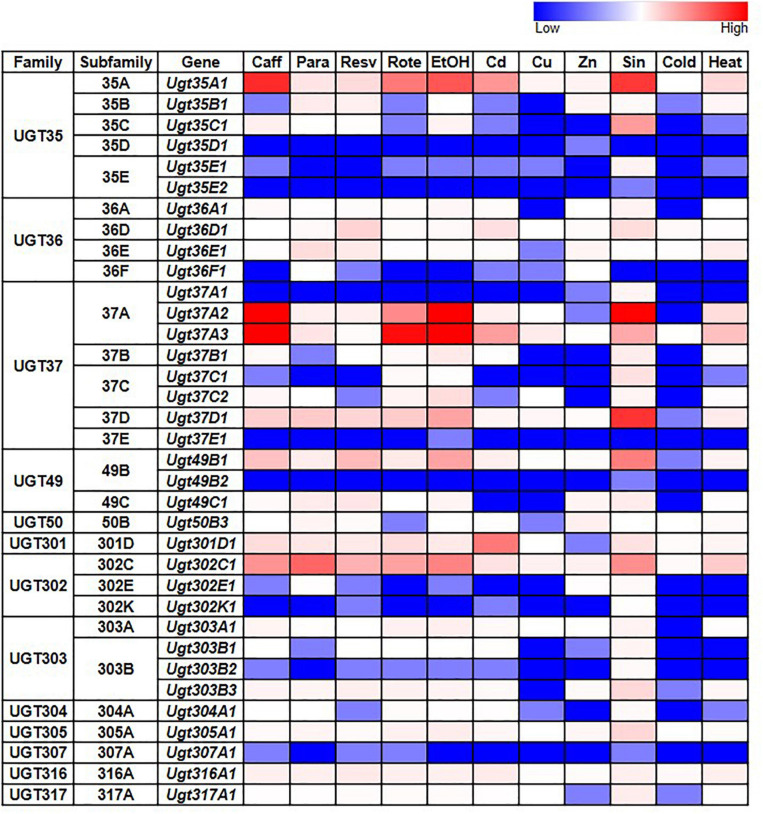
Expression of *D. melanogaster* UGT genes in wild type larvae/adults after various treatments (modENCODE; Brown et al., 2014). Caff: starved L3 larvae were fed 5 mg/ml caffeine for 4 h; Para: 3-day-old adults were fed 10 mM paraquat for 24 h; Resv: 2-day-old adults were fed 100 μM resveratrol continuously for 10 days; Rote: Feeding L3 larvae were fed 2 μg/ml rotenone for 6 h; EtOH: L3 larvae were treated with 5% ethanol; Cd: starved L3 larvae were fed 0.05 mM CdCl_2_ for 12 h; Cu: starved L3 larvae were fed 0.5 mM CuSO_4_ for 12 h; Zn: 2-day-old adults were fed 4.5 mM ZnCl^2^ for 48 h; Sin: L3 larvae were exposed to *Sindbis* virus; Cold: 4-day-old adults were kept at 0°C for 9 h, followed by 2 h of recovery at 25°C; Heat: 4-day-old adults were kept at 36°C for 1 h followed by a 30-min recovery at 25°C. See Supplementary Table 3 for details.

Given the documented role of some UGTs in detoxification, we also examined whether *D. melanogaster* UGT gene expression is induced after exposure to various environmental and chemical treatments by examining RNAseq data generated by the modENCODE project (Brown et al., 2014) – the full dataset is in Supplementary Table 3; representative subsets are in Figure 3. The expression of most UGT genes is not upregulated in response to the majority of treatments. However, six genes from four different UGT families (*Ugt35A1*, *Ugt37A2*, *Ugt37A3*, *Ugt37D1*, *Ugt49B1*, and *Ugt302C1*) clearly show upregulated expression in response to the addition of caffeine, rotenone or ethanol to the diet, or exposure to *Sindbis* virus. On the other hand, certain treatments, including cold exposure and increased dietary copper or zinc, have no/little effect on the expression of any UGT gene.

### UGT Genes in Other *Drosophila* Species

**FIGURE 4:**
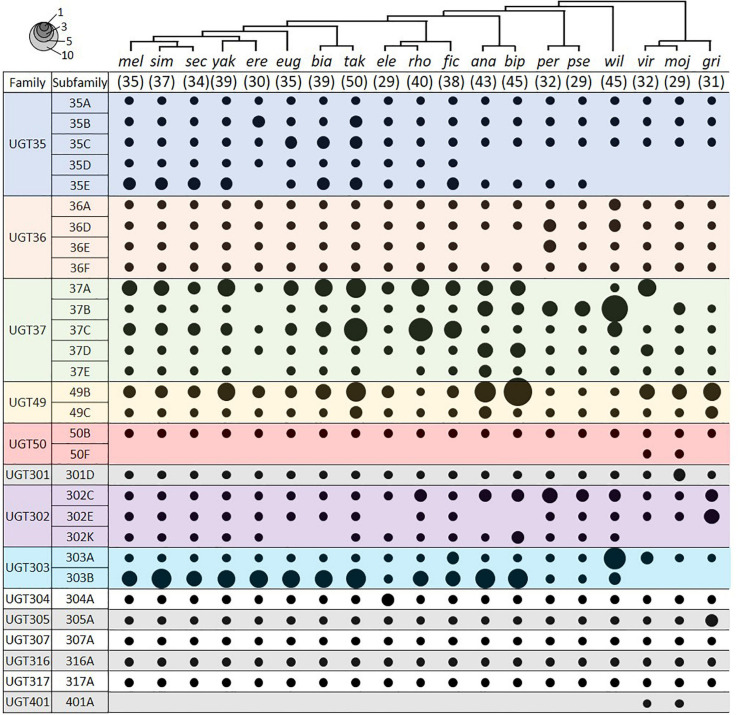
UGT orthologs in 19 *Drosophila* species. Circle size represents the number of genes in the indicated group. The species tree is adapted from Seetharam and Stuart (2013). The number in parenthesis under the tree represents the total number of UGT genes in the given species. Species names refer to *D. melanogaster, D. simulans, D. sechellia, D. yakuba, D. erecta, D. eugracilis, D. biarmipes, D. takahashii, D. elegans, D. rhopaloa, D. ficusphila, D. ananassae, D. bipectinata, D. persimilis, D. pseudoobscura, D. willistoni, D. virilis, D. mojavensis*, and *D. grimshawi*. See Supplementary Table 4 for details.

We identified UGT genes in 18 additional *Drosophila* species and deduced their orthologous relationships to the *D. melanogaster* genes (Figure 4; see section “Materials and Methods”). The total number of UGT genes per genome varies from 29 in *D. elegans*, *D. pseudoobscura*, and *D. mojavensis*, to 50 in *D. takahashii*. Some UGT families have been preserved, whereas others have been multiplied or lost through evolution (Figure 4 and Supplementary Table 4). The conserved UGT families are mostly single-member families, such as UGT50, UGT301, UGT304, UGT305, UGT307, UGT316, and UGT317, and show little or no gene additions/losses. The other UGT families comprising multiple genes show variable gene additions or losses in the different species (Supplementary Table 4). One of the most fluctuating families is UGT37: there are 8 gene members in *D. melanogaster*, but the number increases up to double (16 genes) in *D. rhopaloa* followed by *D. willistoni* (15 genes), and decreases down to half (4 genes) in *D. erecta* and *D. grimshawi*. The UGT49 family also shows a high degree of species difference: there are 3 gene members in *D. melanogaster*, but the number increases up to 11 in *D. bipectinata* followed by 8 in *D. ananassae*.

Two UGTs that are not orthologous with any *D. melanogaster* UGTs were detected in both *D. virilis* and *D. mojavensis*. One pair is an additional member of the UGT50 family, named as the UGT50F subfamily in this study. The other pair defines a new UGT family, named here as *Ugt401A*. By BLAST search in NCBI, additional UGT50F members were found in three other species not included in this study (*D. arizonae*, *D. navojoa*, and *D. hydei*), whereas orthologs of UGT401A were present in seven other species (*D. arizonae*, *D. navojoa*, *D. hydei*, *D. novamexicana*, *D. albomicans*, *D. innubila*, and *D. busckii)*. As all of these species form a distant group (“*repleta-virilis*” group) from *D. melanogaster*, the UGT401A genes might have been lost after divergence of two sub-genera, *Sophophora* and *Drosophila*, or newly emerged in this group, probably playing a unique role.

Further comparative analyses amongst *Drosophila* and related species will become possible as additional genomes are sequenced and annotation pipelines are improved. This will likely reveal other interesting evolutionary patterns. For example, our preliminary analysis of the genome (Gloss et al., 2019) and transcriptome (Whiteman et al., 2012) of *Scaptomyza flava*, a herbivorous leaf-mining species belonging to the Drosophilidae family (Whiteman et al., 2011), reveals that this species has only 23 UGT genes (data not shown), the smallest number among the species surveyed in this study.

## Conclusion and Perspectives

The UGT gene family is one of the largest in the glycosyltransferase (GT) superfamily (EC:2.4.x.y). Since the pioneering work by Myers and Smith (1954), a large body of research outcomes on insect UGTs has been accumulated (Nagare et al., 2020). However, their molecular characteristics are less defined compared to the other detoxification enzymes, such as cytochrome P450s, glutathione S-transferases, and carboxylesterases. One of the reasons is that UGT genes have been incorrectly annotated in many genome sequencing projects. The nomenclature updates and genome-wide analyses of the *D. melanogaster* UGTs in this study will facilitate future work and communication in this growing research domain.

Conjugation with sugar residues changes the properties of aglycone substrate molecules by decreasing the reactivity of functional groups and by increasing solubility, thereby combating toxic xenobiotics (Heckel, 2018). The six genes (*Ugt35A1*, *Ugt37A2*, *Ugt37A3*, *Ugt37D1*, *Ugt49B1*, and *Ugt302C1*) upregulated upon noxious treatments would be the most promising elements potentially responsible for metabolic detoxification of xenobiotics. On the other hand, UGT genes that are highly expressed in specific tissues (e.g., *Ugt35B1, Ugt50B3*, and *Ugt305A1*) are likely to play important physiological roles by conjugating endogenous molecules. Two olfactory UGTs (*Ugt35B1* and *Ugt36E1*) may give a new insight on management of the congeneric pest species, *D. suzukii*. Much more remains to be discovered in relation to the molecular functions of UGTs in sclerotization, pigmentation, immunity and other processes.

## Materials and Methods

### *Drosophila* Genomic Data

Genomic data for *D. melanogaster* UGTs were obtained from FlyBase (flybase.org; Thurmond et al., 2019) using release FB2020_05, which includes *D. melanogaster* genome annotation R6.36. Genomic data for other *Drosophila* species were obtained from NCBI – sequence assemblies and annotation versions are given in Supplementary Table 4. Supplementary Data File 1 contains all *Drosophila* UGT protein sequences in fasta format. The signal peptides and transmembrane domains shown in Supplementary Table 1 were predicted by SignalP-5.0 Server^3^ and TMHMM Server v. 2.0^4^, respectively.

### Phylogenetic Analysis

Deduced amino acid sequences of 35 *D. melanogaster* UGT sequences were aligned by ClustalW and a consensus phylogenetic tree was constructed using the Maximum Likelihood method and JTT matrix-based model with 1,000 bootstrappings. As an outgroup, *fringe* (CG10580), an *N*-acetylglucosaminyltransferase, was used. Evolutionary analyses were conducted in MEGA X (Kumar et al., 2018). The species phylogenetic tree of *Drosophila* used in Figure 4 was adapted from that in (Seetharam and Stuart, 2013).

### *D. melanogaster* UGT Expression Data

Tissue expression (RNAseq) data were downloaded from FlyAtlas2 (^5^ Leader et al., 2018). Gene FPKM (Fragments Per Kilobase of transcript per Million mapped reads) and Enrichment (measuring the abundance of a gene in a particular tissue relative to that in the whole fly) data for adult males, adult females and larvae were downloaded as TSV files and processed in Excel (Supplementary Table 2). FPKM data for adult males and larvae are presented in Figure 2.

modENCODE treatment expression (RNAseq) data (Brown et al., 2014) for were obtained from FlyBase (^6^ Thurmond et al., 2019) using the Batch Download tool operated on the gene_rpkm_report precomputed file. Data were processed in Excel (Supplementary Table 3) and a subset of representative data are presented in Figure 3.

### Identification of UGT Genes in Other *Drosophila* Species

UDP-glycosyltransferases genes in 18 non-*melanogaster* species were additionally identified, which are *D. ananassae* (taxID: 7217), *D. biarmipes* (taxID: 125945), *D. bipectinata* (taxID: 42026), *D. elegans* (taxID: 30023), *D. erecta* (taxID: 7220), *D. eugracilis* (taxID: 29029), *D. ficusphila* (taxID: 30025), *D. grimshawi* (taxID: 7222), *D. mojavensis* (taxID: 7230), *D. persimilis* (taxID: 7234), *D. pseudoobscura* (taxID: 7237), *D. rhopaloa* (taxID: 1041015), *D. sechellia* (taxID: 7238), *D. simulans* (taxID: 7240), *D. takahashii* (taxID: 29030), *D. virilis* (taxID: 7244), *D. willistoni* (taxID: 7260), and *D. yakuba* (taxID: 7245), in alphabetic order. All the UGTs were classified into families/subfamilies using three complementary approaches. First, *D. melanogaster* UGT gene/protein sequences were used as queries of other *Drosophila* genomes available at NCBI using NCBI BLAST. In case of multiple genes in a same gene family, genomic locations were further compared with those of *D. melanogaster* to confirm the orthologous families/subfamilies they belong. Second, the InterPro database (release 82.0;^7^ Mitchell et al., 2019) was queried using the InterPro signature “UDP-glucuronosyl/UDP-glucosyltransferase” (IPR002213), which is diagnostic of UGT proteins, within the *Drosophila* genus (taxon ID 7215). Third, the OrthoDB v10.1 database (^8^ Kriventseva et al., 2019) was also queried using the IPR002213 signature within the *Drosophila* genus (taxon ID 7215) to identify orthologous groups comprising UGT genes. In addition, OrthoDB v9.1 data were obtained via *D. melanogaster* orthology data present in FlyBase (FB2020_05), primarily to obtain OrthoDB groupings for genes in *Drosophila* species absent from v10.1 (*D. simulans*, *D. sechellia*, *D. persimilis*). Data were cross-referenced using the NCBI gene IDs, FlyBase gene IDs and/or UniProt accessions present in each database, and the integrated data are shown in Supplementary Table 4. There is a large (mainly 1:1) agreement between the UGT subfamilies defined by the UGT Nomenclature Committee and the orthologous groups defined by OrthoDB (see Supplementary Table 5 for details). Note that several UGT gene models are incorrectly annotated at FlyBase/NCBI, e.g., some gene models need to be split, others need to be merged, others require extending (see Supplementary Table 4 for details). Also note that all non-*melanogaster* gene models and IDs have been retired from FlyBase and are now annotated and maintained by the NCBI (see the FB2018_06 and FB2020_03 release notes^9^). However, since archived non-*melanogaster* data are still present in FlyBase, and FlyBase IDs/symbols are still present in many databases, FlyBase gene IDs for the non-*melanogaster* species are included in Supplementary Table 4.

## Data Availability Statement

The raw data supporting the conclusions of this article will be made available by the authors, without undue reservation.

## Author Contributions

S-JA and SM designed the research, performed the analyses, evaluated the data, interpreted the results, and wrote the manuscript.

## Conflict of Interest

The authors declare that the research was conducted in the absence of any commercial or financial relationships that could be construed as a potential conflict of interest.

## Footnotes

1 https://prime.vetmed.wsu.edu/resources/udp-glucuronsyltransferase-homepage/current-nomenclature

2 https://flybase.org/reports/FBgg0000797

3 http://www.cbs.dtu.dk/services/SignalP-5.0

4 http://www.cbs.dtu.dk/services/TMHMM

5 http://flyatlas.gla.ac.uk/FlyAtlas2/index.html

6 http://flybase.org

7 https://www.ebi.ac.uk/interpro

8 https://www.orthodb.org

9 https://flybase.org/static/new-this-release
