# Supplementary material for: The UDP-Glycosyltransferase Family in Drosophila melanogaster: Nomenclature Update, Gene Expression and Phylogenetic Analysis
Source: Front Physiol. 2021 Mar 17;12:648481. doi: 10.3389/fphys.2021.648481 (PMC8010143; doi:10.3389/fphys.2021.648481)
Supplement: Supplementary file 7 [file Data_Sheet_2.PDF]

Supplementary Figures

(A) Chromosome 2R

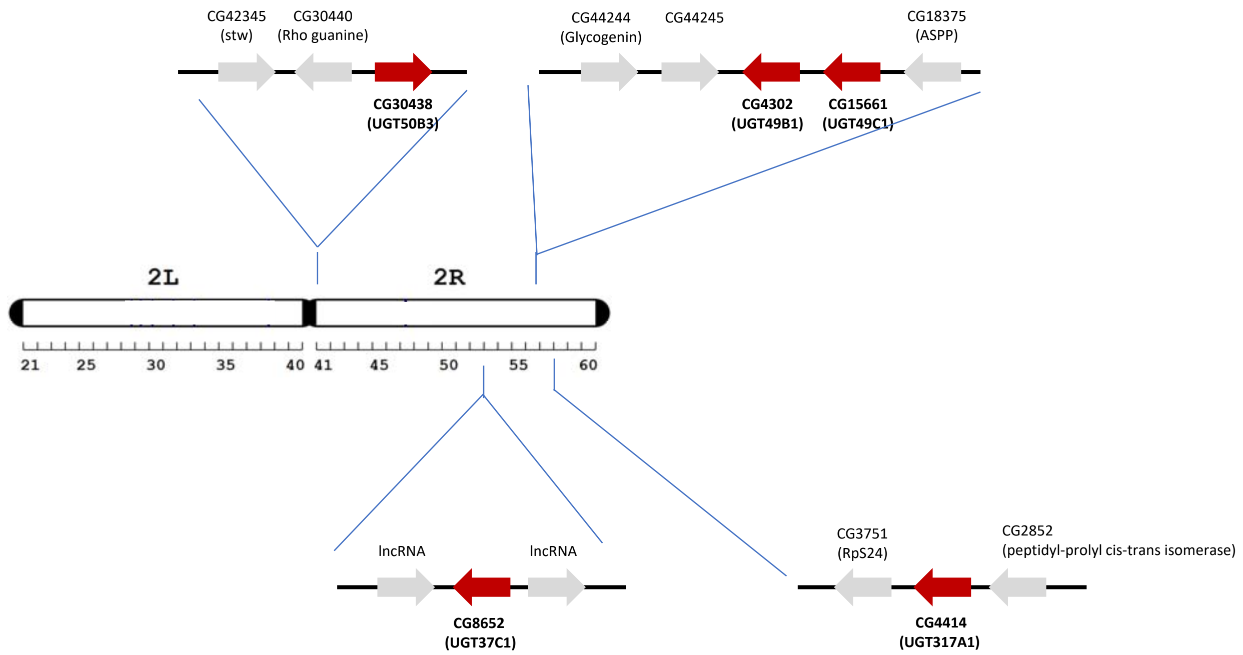

(B) Chromosome 2L

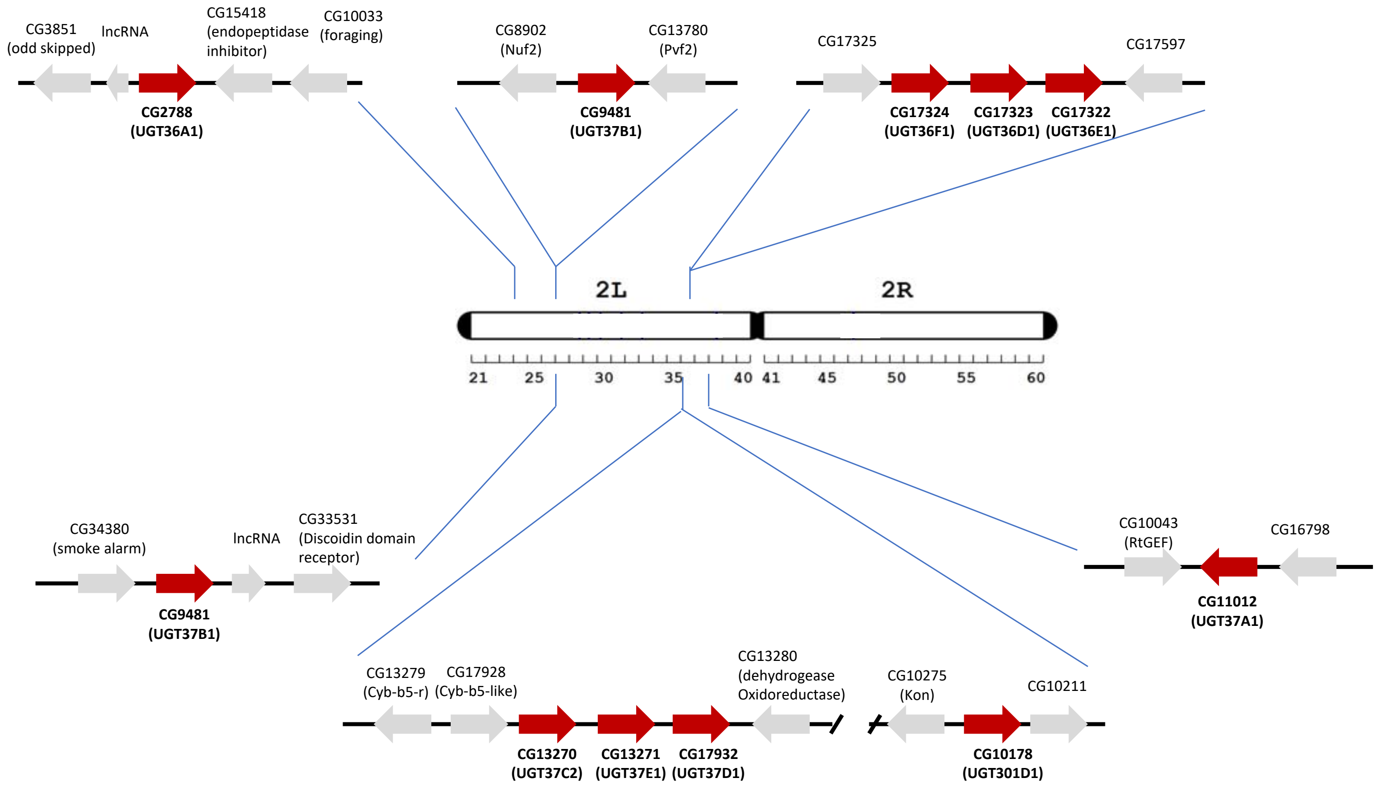

(C) Chromosome 3R

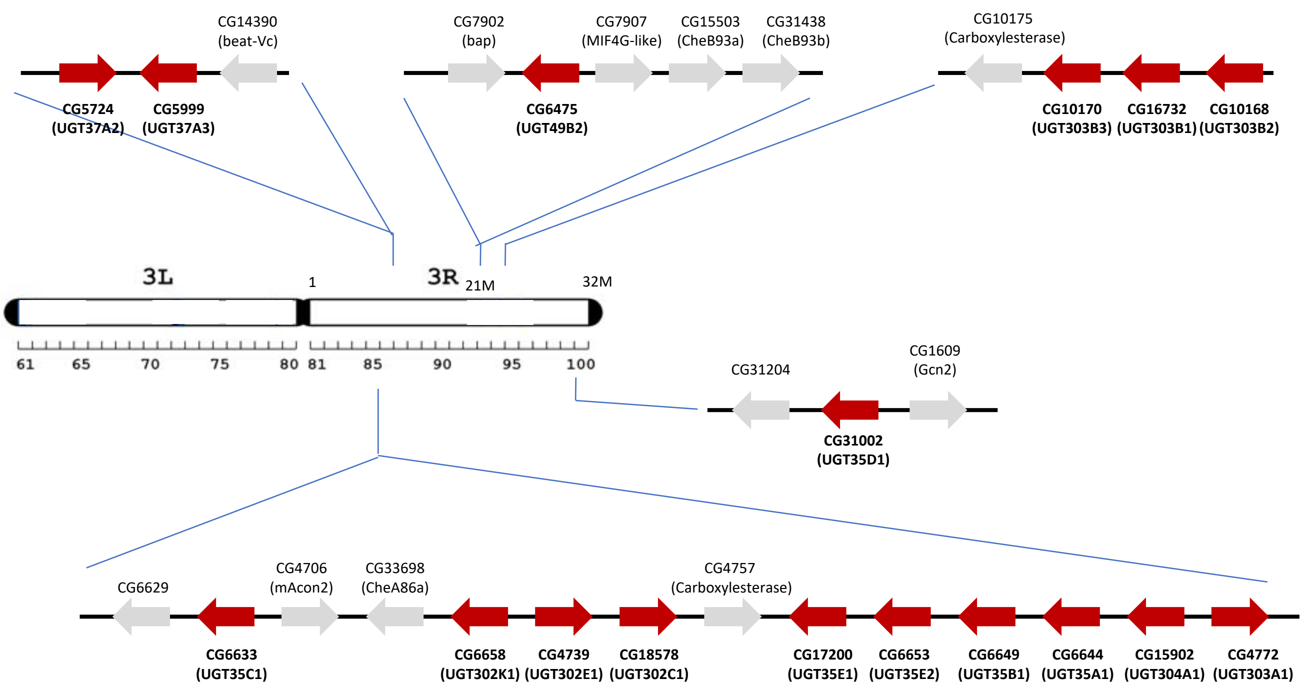

(D) Chromosome 3L

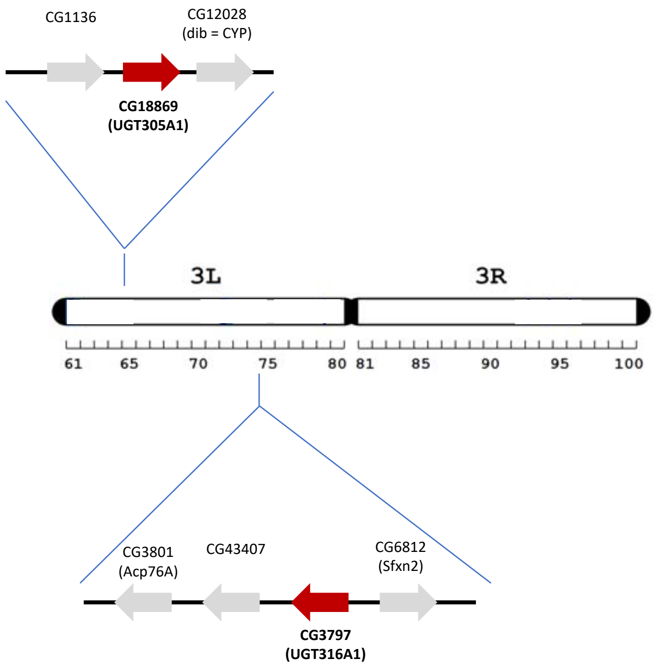

Figure S1. Distribution of UDP-glycosyltransferase genes on the Drosophila melanogaster chromosomes. All of 35 UGT genes are found in two major autosomes (chromosomes 2 with 16 genes and chromosome 3 with 19 genes). The numbers under the chromosome diagrams depict the cytogenetic locations. UGT genes are represented in red and flanking non-UGT genes are in gray in the enlarged scaffolds. Arrow indicates gene orientation.

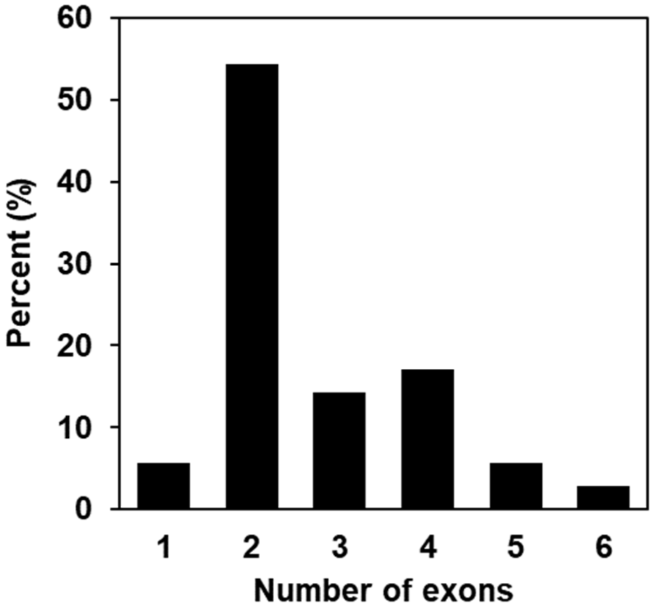

Figure S2. Number of exons of *Drosophila melanogaster* UGT genes. Exons are counted only in protein coding region.

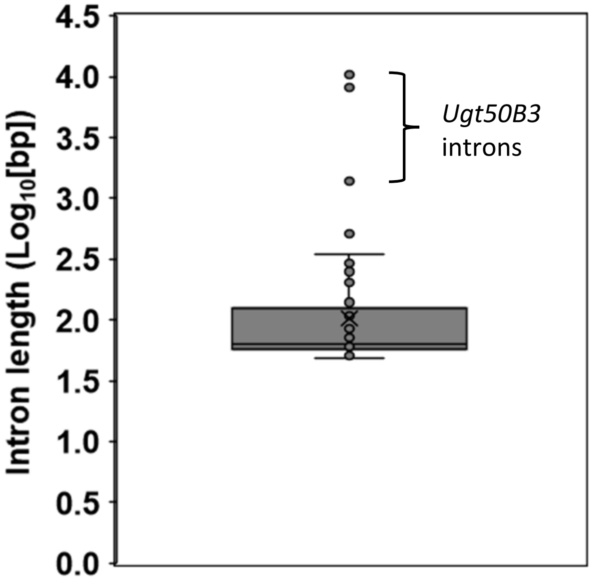

Figure S3. Length of introns of *Drosophila melanogaster* UGT genes. *Ugt50B3* has three long introns among six introns in total.

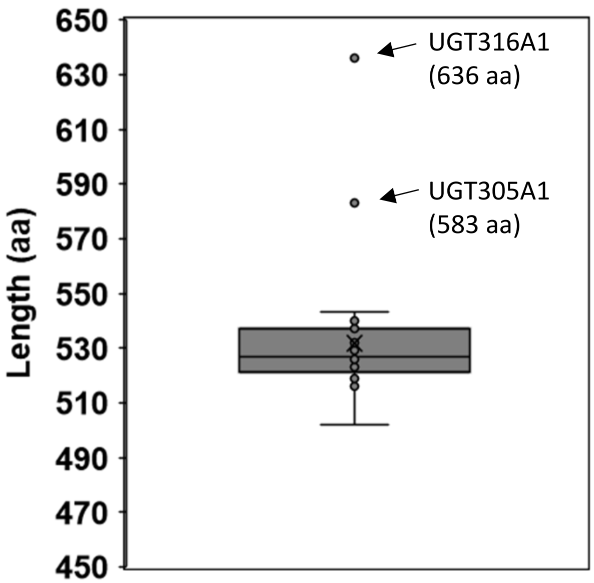

Figure S4. Length of deduced protein sequences of *Drosophila melanogaster* UGT genes. Average length is 531 aa. Two outliers are indicated (*Ugt316A1* and *Ugt305a1*).

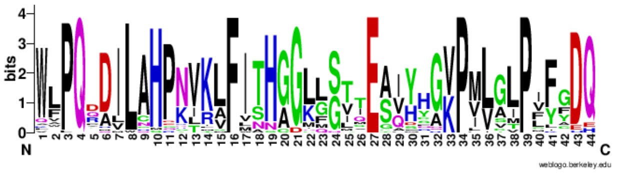

```
Ugt35A1    WFPQPDILAHPNVKLFITHGGLLSTIESIYFGKPILGLPIFYDQ
Ugt35B1    WFPQPDILAHPKVKLFITHGGLLSTIESIHHGKPVLGLPFFYDQ
Ugt35C1    WYPQPDILAHPNVKLFISHGGLLSSTESVYFGKPILGLPCFYDQ
Ugt35D1    WFPQQAILAHPNVKLFITHGGLLSTIESIHHGKPMLGLPCLFDQ
Ugt35E1    WLPQQDLLAHPKVKLFITHGGMQSTIESIHYGKPMLGLPFFYDQ
Ugt35E2    WFPQQDILAHPNVKLFITHGGLQSTVECIHRGVPMLGLPFFYDQ
Ugt36A1    WAPQLALLCHPKVKLFWSHGGLLGTTESVHCGKPLLVTPIYGDQ
Ugt36D1    WLPQRDILCHPNVKVFMSHGGLMGTSEAAYCGVPVVATPMYGDQ
Ugt36E1    WLPQRDILAHPNLKVFMSHGGLMGTTEAVSSAVPIVGVPIYGDQ
Ugt36F1    WLPQRDLLCHPKIRAFISHGGLLGTTEAIHCGVPMLVTPFYGDQ
Ugt37A1    WLPQDDILAHPSVKLFINHAGKGGISEAQYHGKPMLSLPVFGDQ
Ugt37A2    WLPQDDILAHPNIKLFINHAGKGGITEAQYHGKPMLSLPVFGDQ
Ugt37A3    WLPQDDILAHPNIKLFINHAGKGGITESQYHGKPMLSLPVFADQ
Ugt37B1    WVPQVDVLAHPNITLFITHAGKGGLTEAQYHGKPMLALPVFGDQ
Ugt37C1    WLPQDDILAHPNTKLFVTHAGKGSITESQYHGVPMVALPIFGDH
Ugt37C2    WLPQDDILAHPNTKLFITHAGKGGITEAQYHGVPMVALPIFGDQ
Ugt37D1    WLPQDDILAHPKLKLFITHAGKGGVAEAQYHGVPMLALPVFADQ
Ugt37E1    WLPQDDILAHPNTKLFITHAGKGGVAEAQYHGVPMVALPIFGDQ
Ugt49B1    WLPQGDILAHPNVKVFIAHGGLFGTQEAVYNGVPILGMPVYCDQ
Ugt49B2    WLPQADILAHPNVKVFIAHGGLFGMQEAVYHAVPVLGMPFYFDQ
Ugt49C1    WLPQADILAHRHVKVFITHGGLFGTQEGVHYAVPMLGIPFYCDQ
Ugt50B3    WLPQQDILGHPKLRAFVTHGGLLSMFETVYHGVPVVTMPVFCDH
Ugt301D1   WMPQNDILAHPNVKLFITHGGIFGTQEGIYWGVPMLCVPLYGDQ
Ugt302C1   WFPQDDILAHDNVLAFITHGGLLSTTESIYHRKPFVGIPIFGDQ
Ugt302E1   WLPQDDILAHEKVIAFITHGGLLSTMESIYHGKPVVGIPFFGDQ
Ugt302K1   WFPQTDILAHPKIMAFVTHGGMLSTTESIYHAKPVIGLPIFSDQ
Ugt303A1   WFPQRAILNHPNVKLFITHAGLLSLIESVHYAVPLLCIPLFYDQ
Ugt303B1   QAPQRHILNHPNVRLFITNGGLLSVIEAVDSGVPMLGLPMFFDQ
Ugt303B2   KVPQRMVLAHPNLRLFITHGGLQSVMEAIDNGVPMLGLPLFFDQ
Ugt303B3   QPPQRAVLAHPNTRLFITNGGLLSVMEAVYSGVPILGLPVFFDQ
Ugt304A1   LLPQQAILAHPNVKLFISHCGMMSVIEAAYYAKPVLGLPSFFDQ
Ugt305A1   VSLQQDILAMQYVKGFLNHGDSFSLQEAIHYGVPVVVLPLKLEE
Ugt307A1   WWPQTQMWQSGKVRLFITHGGKGSVSEALFYGVPMLGLPLIGDQ
Ugt316A1   WWPQQAILHHPQVKMFISSCGQLSVWESISGQKPVLAIPILAEQ
Ugt317A1   AVPQIDILAHPRVKAFLTNGDLLNLQEGIMRNVPILGLPLFQHE
```

Figure S5. Alignment of the signature sequence of 44 amino acids from *Drosophila melanogaster* UGTs, which is responsible for binding the UDP moiety of the sugar donor. Threshold for shading was set 60%. The sequence logo above the alignment was created by WebLogo (https://weblogo.berkeley.edu).
